# President’s Address

**Published:** 1888-04

**Authors:** W. N. Morrison

**Affiliations:** St. Louis, Mo.


### THE DENTAL REGISTER.

Vol. XLII.] APRIL, 1888. [No. 4.

### COMMUNICATIONS.

### President's Address.

W. N. MORRISON, D.D.S., ST. LOUIS, MO.

[Read before the Mississippi Valley Dental Association, held at Cincinnati, O., March 7, 1888.]

*Gentlemen of the Mississippi Valley Dental Association:*

It affords me great pleasure to be with you on the forty-fourth anniversary of this grand old society, and being your second best man, I must ask your generous indulgence for all short comings and your hearty support and assistance to make this meeting equal to its predecessors—then my highest ambition will be gratified. I will not detain you long as your committee has arranged an admirable program which will occupy all your time profitably when fully carried out.

Let us have the least politics possible. Our papers and speeches should be short and concise. The day for long-winded productions is past; life is too short to sit through them or read them ; we must have things in telegramic proportions if we wish them to be read or remembered. Let us have contrifugal machine articles and speeches in societies which give us the cream without taking so much blue milk. While the scientific investigation of the cause of decay of the teeth goes on, we should not sit as idle lookers on till the question is decided. We know that the mouths kept cleanest require the least of our skill, and be the destruction caused by chemical, vegetable or animal agents, singly or combined, clean mouths are not favorable for their action. Beautiful fillings and artistic restorations are intrusted to uneducated patients who, stimulated by an excavator, will promise to do anything exacted while in the dental chair—they go out and neglect their duty and return reluctantly when forced to come, bringing the information for our special benefit, that fillings do not preserve the teeth from decay.

Dentists are daily committing the error of not instructing their patients in regard to the proper methods of cleansing their mouths,—brushing, picking, rinsing with warm water after meals and at night before going to bed. Our observations must show that people who do these things faithfully, have little or no occasion for dental operations. It is astonishing what ignorance exists among people of all classes and conditions, as to what cleanliness of the mouth means. They will tell you frankly that they do not brush their mouths as well as they ought to, for they did not know they were going to be examined and when you looked, *you really thought so*, and the second thought was, *probably not for a month.* Cleanly habits are part of an individual's education and can only be formed in childhood. Too much care cannot be bestowed upon the subject for the little ones. Each individual must see it thoroughly done—have it done for him, and experience having it well rubbed in with a brush. Not much dentrifice of any kind is needed.—small quill toothpicks are best, narrow strips of rubber dam for spaces the quill will not clean. Water used frequently for rinsing, with a motion of the tongue upon all the surfaces of the teeth and gums, lingual, palatal, labial and buccal. So much for preventive dentistry which should be our highest aim.

Render every thing to its most useful and healthy condition in the shape of a tooth or root,—keep on occluding tooth to every tooth. After extracting a tooth or root through mistaken judgment, restore the lost part and be enough ashamed of the operation to acknowledge it and replant or transplant another in its place. Leave deciduous teeth in the arch to the last moment for their use in mastication, to fill up the arch and that they may contribute their own substance to build up the new osseous structures—the crowns acting as caps over the new crowns. Irregularities are not so frequent where these teeth are allowed to remain ; make space with wedges between the teeth in crowded arches of growing children to prevent decay.

Tropical fruits, particularly those of the citrous family, are showing their effects on the teeth. The Americans as a people are extremists and carry every thing to excess, as is well shown by their diet. Lemon and hot water in the morning, lemon with your fish or steak for breakfast. For dinner, lemon with your oysters on the shell, lemon in your soups, lemon with many other dishes that follow, lemon cooked up with eggs and sugar, custards and pies, and lemon in the finger-bowl. Lemonade and often a plain lemon straight; and when a little out of sorts, a hot lemonade at night on going to bed.

Sweets constitute such an important part of our diet. Children are bribed to do plain duties, by promises of more sweetmeats. Drinks which would have been sickeningly sweet to a palate of years ago, are daily demanded by children of our day, Syrups to excess are used daily in all families. Milk and eggs are ruined in their healthful simplicity by the addition of sugar and lemons; and other mixtures are made that deprive the teeth of use in mastication and furnish the secretions of an abnormal quality favoring destruction by decay.

I have watched with much interest and sadness, the teeth of children in families where much sugar was used and invariably their teeth were bad. One family, relatives of mine, used four barrels of sugar a year. When adult life was reached, there was not one mouth with the full quota of teeth—all had extensive fillings, and two of the young ladies have their mouths full of crockery substitutes. Employes of long service in confectionery shops and candy factories are also affected in the same manner. It is the excessive use of these articles, simple in themselves, which is doing the irreparable hereditary damage.

How humiliating would be an admission that an American of mixed national parentage could not grow jaw enough to accommodate thirty-two teeth. How humiliating is the fact, that of the twenty-eight or less teeth remaining by permission of the zealous dentist, their durability and use are constantly being jeopardized by a diet of concentrated chemical form. The patient's negligence and an appetite for this kind of food requiring no mastication, needing no insalivation, but which digests in the mouth, dissolves the teeth that dare not insert themselves in such toothsome morsels.

What a shame that condiments constitute such a portion of the diet in the nineteenth century. If we keep on at this rate, in generations to come, the most concentrated acids or alkalies will be barely able to tickle the palate or warm the stomach of a young lady of eighteen years, whose teeth are of insoluble crockery—her ancestors having had the same.

In conclusion, I would like to have said something of the materials to be used in fillings. We have all the elements in nature's realm at our command. Let us use anything to preserve a tooth, if only temporarily—even a substance once proscribed by this old society in its younger days—amalgam.

I trust the dentist of the future will have skill and accumulated knowledge at his command not dreamed of by the most advanced of this day.

### My Experience with the Dental Pulp.

BY WM. A. PEASE, DAYTON, O.

(Read before the Mississippi Valley Dental Association.)

